# Clinical and neurophysiological effects of bilateral repetitive transcranial magnetic stimulation and EEG-guided neurofeedback in Parkinson’s disease: a randomized, four-arm controlled trial

**DOI:** 10.1186/s12984-024-01427-5

**Published:** 2024-08-05

**Authors:** Juan Pablo Romero, Marcos Moreno-Verdú, Aida Arroyo-Ferrer, J. Ignacio Serrano, Jaime Herreros-Rodríguez, Juan García-Caldentey, Eduardo Rocon de Lima, María Dolores del Castillo

**Affiliations:** 1 Brain Injury and Movement Disorders Neurorehabilitation Group (GINDAT), Institute of Life Sciences, Universidad Francisco de Vitoria, Pozuelo de Alarcón, Spain; 2 Facultad de Ciencias Experimentales, Universidad Francisco de Vitoria, Pozuelo de Alarcón, Spain; 3 Brain Damage Unit, Hospital Beata María Ana, Madrid, Spain; 4 Brain, Action, and Skill Laboratory (BAS-Lab), Institute of Neuroscience (Cognition and Systems Division), UC Louvain, Av. Mounier 54 (Claude Bernard), Floor +2, Office 0430, Woluwe-Saint-Lambert, 1200 Belgium; 5 Neural and Cognitive Engineering Group, Centre for Automation and Robotics, Spanish National Research Council, Madrid, Spain; 6 Department of Neurology, University Hospital Infanta Leonor, Madrid, Spain; 7 Department of Neurology, Hospital Quirón Palmaplanas, Palma de Mallorca, Spain

**Keywords:** Parkinson’s disease, Non-invasive neuromodulation, Repetitive transcranial magnetic stimulation, Neurofeedback, EEG, Motor symptoms

## Abstract

**Background:**

Repetitive Transcranial Magnetic Stimulation (rTMS) and EEG-guided neurofeedback techniques can reduce motor symptoms in Parkinson’s disease (PD). However, the effects of their combination are unknown. Our objective was to determine the immediate and short-term effects on motor and non-motor symptoms, and neurophysiological measures, of rTMS and EEG-guided neurofeedback, alone or combined, compared to no intervention, in people with PD.

**Methods:**

A randomized, single-blinded controlled trial with 4 arms was conducted. Group A received eight bilateral, high-frequency (10 Hz) rTMS sessions over the Primary Motor Cortices; Group B received eight 30-minute EEG-guided neurofeedback sessions focused on reducing average bilateral alpha and beta bands; Group C received a combination of A and B; Group D did not receive any therapy. The primary outcome measure was the UPDRS-III at post-intervention and two weeks later. Secondary outcomes were functional mobility, limits of stability, depression, health-related quality-of-life and cortical silent periods. Treatment effects were obtained by longitudinal analysis of covariance mixed-effects models.

**Results:**

Forty people with PD participated (27 males, age = 63 ± 8.26 years, baseline UPDRS-III = 15.63 ± 6.99 points, H&Y = 1–3). Group C showed the largest effect on motor symptoms, health-related quality-of-life and cortical silent periods, followed by Group A and Group B. Negligible differences between Groups A-C and Group D for functional mobility or limits of stability were found.

**Conclusions:**

The combination of rTMS and EEG-guided neurofeedback diminished overall motor symptoms and increased quality-of-life, but this was not reflected by changes in functional mobility, postural stability or depression levels.

**Trial registration:**

NCT04017481.

**Supplementary Information:**

The online version contains supplementary material available at 10.1186/s12984-024-01427-5.

## Introduction

Parkinson’s disease (PD) causes a myriad of motor and non-motor symptoms which significantly reduce functionality, decrease independence in everyday activities and impair health-related quality-of-life [1, 2].

Recently, several neuromodulation techniques have been incorporated into the symptomatic management of PD, complementing pharmacologic treatment, primarily based on the administration of dopamine or dopaminergic drugs. Invasive neuromodulation via deep brain stimulation is the most common [3], although it is restricted to specific candidates and is not devoid of secondary effects [4]. On the other hand, non-invasive neuromodulation techniques have also been applied to modify brain connectivity exogenously or endogenously [5]. They are promising techniques as they can be used in wider populations, yet the lack of unified protocols, their limited efficacy and short-lasting after-effects prevent from routinely using them in clinical practice [6].

Exogenous neuromodulation relies on the application of external stimuli without the need for surgery or device implantation. In PD, the most studied technique is repetitive transcranial magnetic stimulation (rTMS), where pulsed magnetic fields are applied to the surface of the skull, generating weak electrical currents in specific brain regions [7]. Depending on the frequency of the stimulation pulses, rTMS can be excitatory (> 5 Hz) or inhibitory (≤ 1 Hz), increasing or decreasing cortical excitability. Several studies have proved the efficacy of excitatory rTMS over the primary motor cortex (M1) on motor symptoms [8, 9], improving the Unified Parkinson’s Disease Rating Scale-III (UPDRS-III) scores between 15 and 49% [10]. Previous studies have also demonstrated the ability of rTMS to increase the Cortical Silent Period (CSP). The CSP is one of the best studied markers of intracortical inhibition and excitability modulation. It is known that in PD, the CSP is usually shortened due to neural dynamics putatively occurring in the subthalamic nucleus [11], and it can be elongated as a result of dopamine administration or subthalamic stimulation [12]. Therefore, the CSP could potentially be used to assess some of the neurophysiological correlates of rTMS interventions in PD.

Among endogenous, non-invasive neuromodulation approaches, neurofeedback (NFB) is a kind of biofeedback which teaches self-control of brain functions by measuring brain waves and providing a feedback signal [13]. By modifying brain activity, NFB aims to produce behavioural changes. NFB can be achieved by several neuroimaging techniques including functional Magnetic Resonance Imaging (fMRI) as the most recent; but electroencephalography-guided (EEG-guided) NFB is probably the most used because of its high temporal resolution, relatively low cost, and wide applicability [14]. In PD, the effects of different types of NFB have been investigated in several trials (see Anil et al. for a recent systematic review) [15]. There are studies suggesting that fMRI-guided NFB by itself may be effective for ameliorating motor symptoms severity in PD (i.e., up to 4.5-point reduction on the UPDRS-III scale) [16], although this is still controversial [17].

Exogenous methods such as rTMS primarily modulate cortical excitability [16], but rTMS has also effects on sensory-motor connectivity [18]. In a similar manner, endogenous techniques like NFB can enhance corticospinal excitability [19] and modify sensory-motor networks encompassing both cortical and subcortical areas [13, 20, 21]. Although the physiological mechanisms of these techniques are not completely understood, given their overlapping effects on the sensory-motor network and motor cortex, we posit that their effects may be synergistically coupled, facilitating more vigorous activation of the sensory-motor network and thus decreasing motor symptoms of PD to a greater extent and with longer lasting effects. Furthermore, since NFB demands cognitive effort and engages attention and other executive functions, and given the documented cognitive effects of rTMS over M1 [22], it seems feasible to prime NFB with rTMS to potentially increase its efficacy on motor and non-motor impairments caused by PD. However, up to date the effects of this combination remain largely unexamined.

There is also a lack of studies investigating the effects of non-invasive neuromodulation techniques on specific motor components (beyond UPDRS-III) such as functional mobility, postural stability, or motor speed, nor non-motor symptoms or concurrent associations with health-related quality-of-life improvements.

The main aim of this multi-arm study was to determine the immediate and short-term effects of exogenous and endogenous neuromodulation techniques, alone or in combination, in comparison to no intervention, in people with PD who are currently receiving pharmacologic therapy. Specifically, we sought to assess the effects of these protocols on a range of PD symptoms, including motor symptom severity, functional mobility, postural stability, motor speed, depression, or health-related quality-of-life. A secondary objective was to analyse whether these neuromodulation approaches had electrophysiological correlates associated with their clinical effects. Our hypothesis was that the combination of rTMS and NFB would demonstrate larger effects on the abovementioned aspects than their isolated use, when compared to no intervention.

## Methods

### Study design

A randomized, parallel group, single-blinded controlled, 4-arm trial was conducted. Reporting followed the CONSORT 2010 guidelines (extension for multi-arm designs) [23]. The study was registered on clinicaltrials.gov (NCT04017481). All participants were informed on the details of the study and gave written informed consent prior to enrolment. The study followed the Declaration of Helsinki (revised in 2013) and an ethical committee of an independent organism approved the study protocol (IRB approval number: 16/37). The trial was conducted at a movement disorders clinic in Madrid, Spain.

### Randomization and recruitment

Permuted block randomization with sequential, stratified recruitment based on UPDRS-III scores was used. Randomization was performed on a computer-generated block allocation schedule by an independent researcher who was not in charge of assessing participants for eligibility. UPDRS-III was administered using a video evaluation by blinded evaluators. After the UPDRS-III assessment, the participant was classified into one of three possible severity groups according to UPDRS-III score, with ranges of 0–36, 37–72 and 73–108 points. In each severity group, the included participants were randomly allocated into four intervention groups using a 1:1:1:1 ratio: groups A (rTMS), B (EEG-guided NFB), C (rTMS + EEG-guided NFB) and D (no intervention). The investigator in charge of concealing allocation included the code of each group in closed opaque envelopes that were opened on the first intervention session of each enrolled participant.

### Participants

We aimed to enrol 40 participants with counterbalanced allocation to the 4 treatment groups.

Eligibility criteria were as follows. Inclusion criteria were: (1) adults (> 18 years), (2) clinical diagnosis of idiopathic PD according to the UK Brain Bank Criteria [24], (3) Hoehn & Yahr stage between I-III, (4) absence of evident motor fluctuations and (5) pharmacological stability (i.e., without changes in antiparkinsonian medication within the last month). Exclusion criteria were: (1) receiving advanced therapies for PD (i.e., apomorphine infusion, duodenal dopamine, deep brain stimulation), (2) epilepsy history or structural alterations in previous neuroimaging studies, (3) mild cognitive impairment or dementia (MoCA < 24 points) [25], (4) suspicion of atypical parkinsonism and (5) diagnosis of any other neurological diseases or severe comorbidity.

### Interventions

#### Group A: bilateral rTMS

Eight rTMS sessions over 2 consecutive weeks were delivered. All sessions were applied at the same time of day in each participant and medication was not suspended during the protocol. All patients were in ON state during the stimulation (1–2 h after the last intake and self-reported in ON state when asked). None of the participants had evident motor fluctuations of wearing off during the study period.

Each session consisted of bilateral, high-frequency (10 Hz) stimulation over both M1 at 80% of resting motor threshold (RMT). A bilateral protocol was chosen as it has shown positive effects on measures of bradykinesia and rigidity [26]. To each M1, 1000 pulses were delivered, divided into 25 trains of 40 pulses each, with a 25-second inter-train interval. The first side stimulated was the M1 corresponding to the most symptomatic hemi-body, with a 5-minute pause between hemispheres.

The site of stimulation was located at the hotspot based on the motor-evoked potential (MEP) response from the abductor pollicis brevis (APB) and the RMT defined as the minimal stimulus intensity required to evoke MEPs of at least 50 µV in 5 of 10 consecutive trials [27]. A refrigerated figure-of-8 70 mm coil connected to a Magstim-Rapid 2 stimulator (Whitland, UK) was used.

#### Group B: EEG-guided neurofeedback (EEG-guided NFB)

Eight EEG-guided NFB sessions over 2 consecutive weeks were delivered. An actiCHamp amplifier (Brain Vision LLC, NC, USA) was used to amplify and digitize EEG data, at a sampling frequency of 512 Hz. EEG activity was recorded from 64 positions with active Ag/AgCl scalp electrodes (actiCAP electrodes, Brain Vision) following the 10–20 international system. Impedance was kept < 10 kΩ. Ground and reference electrodes were placed on Fz and FCz, respectively.

EEG signal processing was conducted using the EEGLab toolbox from Matlab [28]. The continuous EEG signal for each channel was artefact-corrected by the Artifact Subspace Reconstruction algorithm, disabling all parameters except the high-pass filter (0.25–0.75 Hz) and the burst repairing (kurtosis > 5). The signal was then band-pass filtered between 3 and 31 Hz with a Finite Impulse Response filter [29]. Afterwards, channels beyond 5 standard deviations (SD) of the average channel kurtosis were automatically rejected and spherically interpolated. Next, Independent Component Analysis was performed, and artifact-related components were removed according to the Multiple Artifact Rejection Algorithm [30].

**Fig. 1:**
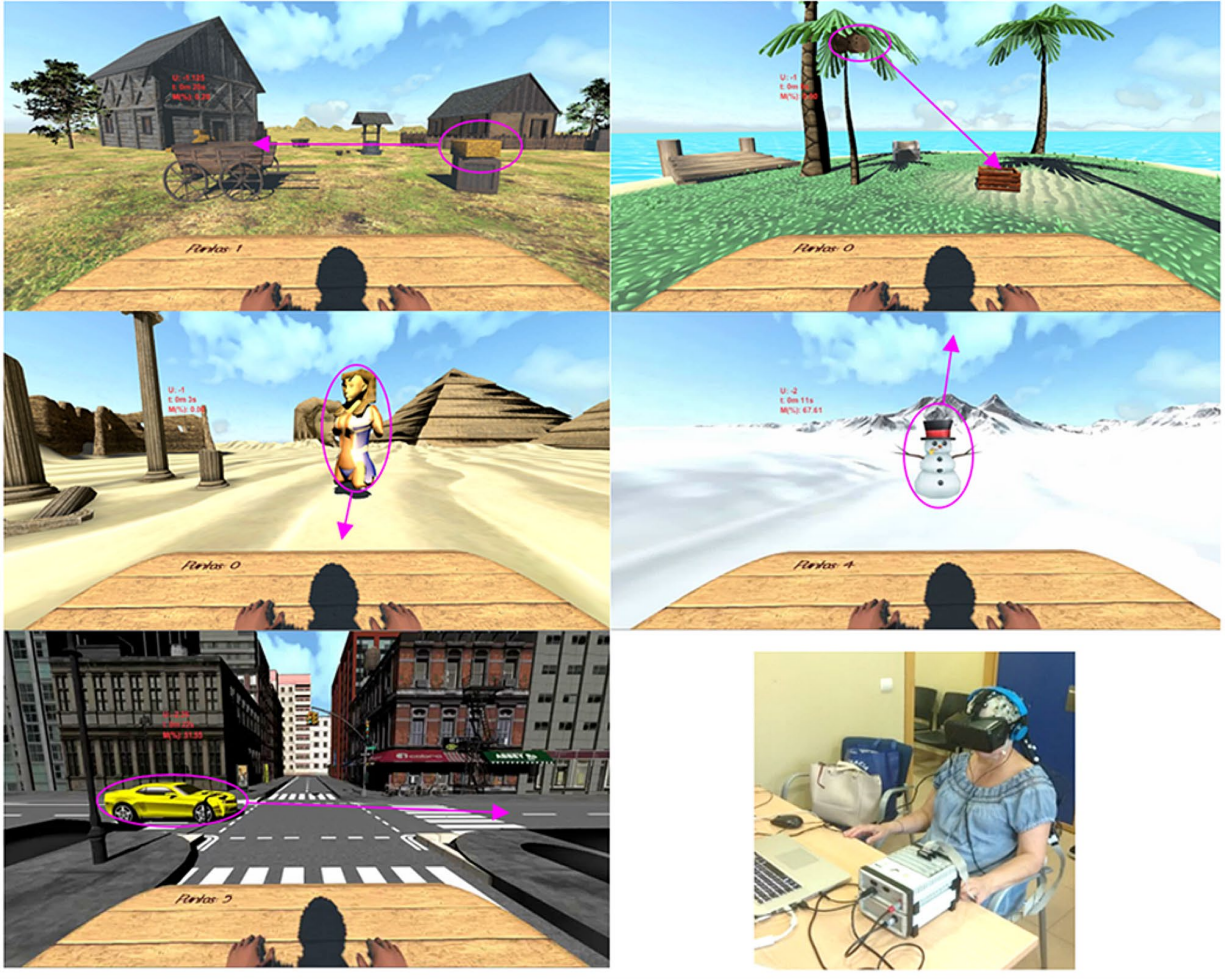
Frames of the different Neurofeedback (NFB) environments designed for the intervention. Objects surrounded by the circles are the moving targets. Arrows indicated the direction and destination of the movement. Bottom right image: Experimental setup of participants, showing the Virtual Reality set up (Oculus^®^ 3D), mounted over the 64-channel EEG cap

Before each session, the EEG signal of the electrodes used for the NFB was captured with 1-minute resting state EEG assessments and the average and SD of the Power Spectral Density (PSD) for all electrodes and the frequency band identified were calculated. Each session consisted of 30 min of EEG-guided NFB using the Oculus^®^ 3D Virtual Reality Glasses (Google, Inc) for visual feedback. The use of immersive VR was motivated by the facilitation of concentration and the ludic contribution of that technology, which leads to an increased motivation and adherence to the intervention [31]. Motivation and concentration were evidenced as enhancers of the NFB effects [32]. The participant intended to move an object in 5 different 6-minute virtual environments with immersive virtual reality and no explicit instruction (Fig. 1).

The target of the neurofeedback intervention was the neuromodulation of the alpha and beta frequencies in the primary motor cortex (M1). These frequencies have been evidenced to be altered (raised) in PD patients [33] and responsive to dopaminergic intake [34]. Consequently, the feedback was produced when achieving a reduction in average bilateral alpha (9–12 Hz) and beta (18–24 Hz) bands [35] in the three central EEG channels C3, Cz, and C4, according to the 10–20 system. Each scenario had a virtual object that moved (feedback) when the average PSD, in windows of 0.5 s with a window step of 3 samples for the identified electrodes, was at least 1 SD lower than the average resting PSD in the identified frequency bands. This threshold was initially set by the therapist so that the participant was able to move the object at least once every 10 s. Then, the threshold was increased daily to the minimum effective. The virtual scenarios were designed with Unity Engine 2018.1.9 (Unity Technologies, USA). EEG signal’s online processing was carried out with NeuroRT Studio (Mensia Technologies Ltd., France). The two software platforms communicated by TCP/IP.

#### Groups C (combined rTMS + EEG-NFB) and D

The protocols were combined for Group C, rTMS followed by EEG-NFB. The rest period between interventions was always 10 min and rTMS was always delivered as a priming method before NFB. For group D, participants did not receive any therapy.

### Outcome measures

Each participant was assessed the day before the start of treatment (T0, pre), the day after the end of treatment (T1, post), and 16 days after (T2, follow-up), by an assessor blinded to group allocation.

#### Primary outcome measure

Motor symptoms severity, assessed with the UPDRS-III, was the primary outcome measure of interest. This assessment was videorecorded and evaluated by three different neurologists experienced in movement disorders. One neurologist (J.P.R) administered it in real time and the other two (J.H.R and J.C.G) through the videos. These two assessors were blinded to the participant’s group and assessment’s time point (i.e., T0, T1 or T2). These scores were the only utilized for statistical analysis.

#### Secondary outcome measures

Functional mobility was assessed with the Timed Up and Go Test (TUG) [36]. In the TUG, the individual get ups from a chair, walks three meters, returns and sits back, and time taken is measured. Higher times indicated worse mobility.

Postural stability was evaluated by Limits of Stability (LOS) from the Biodex Balance System, version 1.08 (Biodex, Shirley, NR). Sampling rate was 100 Hz. Prior to testing, the system adjusted feet setting on the platform according to participant’s height. The body movement to shift the gravity centre (i.e., weight) produced a movement of a cursor from a centred visual target to a blinking target in eight directions. Participants were instructed to go to each target and back to the central as quickly and with less deviation as possible. Each participant completed the test three times. Performance was defined as the percentage that the individual reaches from their theoretical LOS (%LOS) [37]. Lower scores implied poorer stability.

Motor speed was determined with the Finger Tapping speed (FT) [38]. The individual was instructed to press the spacebar of the keyboard with the index finger as quickly as possible and repeatedly. Five 10-second attempts for each hand were collected, starting with the dominant side, in Presentation^®^ (Neurobehavioral Systems, Inc). The average tap-to-tap time was the outcome measure, higher times indicating lower speed.

Depression level was assessed with the Spanish Hamilton Depression Rating Scale (HDRS) [39], higher scores implying lower severity. Participants were classified as having no depression, mild, moderate or severe depression based on HDRS scoring [40]. Health-related quality-of-life was evaluated with the Spanish Parkinson’s disease questionnaire (PDQ-39) [41], where lower scores mean higher quality-of-life. These measures were only evaluated at T0 and T2 based on their sensitivity to change.

Neurophysiological measures were obtained as Cortical Silent Periods (CSP). EMG recording coupled to TMS was collected using 9 mm diameter Ag-AgCl surface electrodes placed on the first dorsal interosseus (FDI) muscle. EMG measurements were amplified 1000 times, filtered with a band pass of 20 Hz – 2.5 kHz using a Digitimer D440-2 amplifier (Digitimer Ltd., UK) and digitized with a CED Micro 1401-3 (Cambridge Electronic Design, UK). All EMG data were pre-processed and analysed using Signal version 6 (Cambridge Electronic Design, UK) [42]. Ten consecutive pulses at 130% RMT were applied during weak voluntary contraction (15–20% of participant’s maximal voluntary contraction). The average MEP-onset until return of voluntary EMG activity time (in milliseconds) was measured for each hemisphere separately [43].

### Statistical analysis

All analyses were implemented in R version 4.1.2 (R Core Team, 2023). The specific packages used are listed in Supplementary Materials. Descriptive statistics was performed by mean and SD for continuous variables and relative frequencies for categorical variables.

Average treatment effects (ATE) for each active treatment (Groups A-C) compared to control (Group D) were obtained through analysis of covariance (ANCOVA) mixed-effects models. Treatment effects were considered fixed and inter-individual variability was considered random (i.e. by-subjects random intercepts). The ATE was obtained as the overall effect across time, and at T1 and T2 separately with baseline covariates using the formula ‘y ~ β_0_ + β_1_*treatment* + β_2_*subject* + β_3_*baseline* + β_4_*time +* β_5_*treatment* x *time*’, as detailed in Twisk et al. [44]. For the HDRS and PDQ39, which were only administered at follow-up, the ANCOVA model could not include random effects nor treatment interactions with time, and therefore they were modelled as ‘y ~ β_0_ + β_1_*treatment* + β_2_*baseline*’. Therefore, all models accounted for baseline measures, which were included as a covariate.

For hypothesis testing (i.e., differences between the active treatment and control), we set alpha = 0.05 for statistical significance and obtained 95% confidence intervals of each ATE for parameter uncertainty. Effect sizes at T1 and T2 were computed using Cohen’s d [45].

Missing data were modelled by multiple imputation with 10 imputed samples and predictive mean matching, in an intention-to-treat analysis, as this is an unbiased method for cluster-randomized controlled trials [46]. We used m = 10 imputed samples as between m = 5–20 is generally recommended under moderate missingness [47]. We assumed the data were missing at random as there was evidence that data were not completely missing at random according to Little’s test (*χ*²(46) = 110, *p* < 0.001). Sensitivity analyses were conducted with other imputation methods (Random sampling from observed values, Unconditional Mean Imputation and Bayesian linear regression) or no imputation (case-wise deletion) to assess the robustness of our imputation choice.

## Results

No changes in any treatment protocol or in the assessment of any outcome measures were performed after the trial commenced.

### Participants’ characteristics and flow

Forty participants (27 males, mean age = 63 ± 8.26 years, 38 right-handed, mean Levodopa Equivalent Daily Dose (LEDD) = 750.29 ± 728.11 mg) were initially enrolled (Table 1). Mean UPDRS-III score was 15.63 ± 6.99 points. Most participants were in Hoehn & Yahr stage II (*n* = 21, 52.5%). Regarding non-motor symptoms, most participants (*n* = 32, 80%) were categorized as having no depression (HDRS < 8 points), a few (*n* = 7, 17.5%) as mild depressive symptoms (HDRS = 8–16 points) and only 1 as moderate depression (HDRS = 17–23 points).

**Table 1:** Sociodemographic and clinical characteristics of the participants at baseline

Variable		Group A^1^ (*n* = 10)	Group B^1^ (*n* = 11)	Group C^1^ (*n* = 10)	Group D^1^ (*n* = 9)	All participants (*n* = 40)
Age, years (mean ± SD)		64.40 ± 6.38	62.18 ± 8.18	59.00 ± 7.62	66.89 ± 9.07	63 ± 8.26
Time with disease, years (mean ± SD)		6.00 ± 3.06	5.91 ± 3.75	5.20 ± 3.16	6.22 ± 4.12	5.83 ± 3.42
UPDRS-III score (mean ± SD)		17.00 ± 8.18	15.55 ± 7.28	15.00 ± 6.39	15.67 ± 10.17	15.8 ± 7.76
LEDD, mg (mean ± SD)		629.45 ± 339.24	537.36 ± 313.67	521.30 ± 291.87	1399.27 ± 1271.3	750.29 ± 728.11
Sex, N of males (%)		7 (70%)	7 (63.63%)	7 (70%)	6 (66.67%)	28 (70%)
Handedness, N of right-handed (%)		10 (100%)	10 (90.9%)	9 (90%)	9 (100%)	38 (95%)
Hoehn & Yahr stage, N (%)	1	1 (10%)	3 (27.27%)	5 (5%)	2 (22.22%)	11 (27.5%)
	1.5	1 (10%)	0 (0%)	0 (0%)	0 (0%)	1 (0.03%)
	2	6 (60%)	5 (45.45%)	4 (40%)	6 (66.67%)	21 (52.5%)
	2.5	1 (10%)	1 (0.09%)	0 (0%)	1 (11.11%)	3 (0.07%)
	3	1 (10%)	2 (18.18%)	1 (10%)	0 (0%)	4 (10%)
Depression severity, N (%)^2^	No depression	7 (70%)	8 (72.73%)	9 (90%)	8 (88.89%)	32 (80%)
	Mild	3 (30%)	3 (27.27%)	0 (0%)	1 (11.11%)	7 (17.5%)
	Moderate	0 (0%)	0 (0%)	1 (10%)	0 (0%)	1 (2.5%)
Left Hemisphere RMT, % (mean ± SD)^3^		60.10 ± 7.06	58.55 ± 6.99	60.00 ± 9.13	56.88 ± 6.51	60.31 ± 5.46
Right Hemisphere RMT, % (mean ± SD)^3^		60.89 ± 5.08	62.09 ± 5.13	59.00 ± 5.16	58.13 ± 6.51	58.97 ± 7.34

^1^Group A received bilateral, high-frequency Repetitive Transcranial Magnetic Stimulation (rTMS). Group B received EEG-guided neurofeedback (EEG-guided neurofeedback). Group C received a combination of rTMS and EEG-guided neurofeedback. Group D did not receive any intervention

^2^Depression severity was assessed by the Hamilton Depression Rating Scale and classified as no depression (0–7 points), mild (8–16 points), moderate (17–23 points) and severe (> 23 points)

^3^In Group D, data is shown for *N* = 8 participants because of technical issues while collecting data from 1 participant

*Abbreviations* LEDD = Levodopa Equivalent Daily Dose; RMT: Resting Motor Threshold; UPDRS-III = Unified Parkinson’s Disease Rating Scale-Part III, motor examination

**Table 2:** Changes in clinical and neurophysiological variables according to group, from baseline (T0) to post-intervention (T1) and follow-up (T2)

Variable	Group^1^		T0	T1	T2
UPDRS-III (points)	A (*n* = 10)		17 ± 8.18	13.2 ± 5.9	12.9 ± 6.66
	B (*n* = 11)		15.55 ± 7.29	13.36 ± 6.28	13.63 ± 5.54
	C (*n* = 10)		15 ± 6.39	10.1 ± 5.82	10.6 ± 4.3
	D (*n* = 9)		14.89 ± 6.88	13.56 ± 5.88	14.56 ± 5.92
TUG (seconds)	A (*n* = 10)		10.99 ± 2.16	11.08 ± 2.52	10.18 ± 1.77
	B (*n* = 11)		10.82 ± 2.32	9.78 ± 1.82	9.86 ± 1.77
	C (*n* = 10)		9.69 ± 1.73	9.39 ± 2.85	9.41 ± 2.88
	D (*n* = 9)		11.53 ± 2.53	10.55 ± 1.56	10.91 ± 2.37
LOS (%)	A (*n* = 10)		42.62 ± 19.72	45.22 ± 14.09	44.15 ± 16.24
	B (*n* = 10)^2^		31.70 ± 12.70	38.35 ± 14.10	39.15 ± 15.21
	C (*n* = 9)		33.02 ± 16.92	34.25 ± 17.12	37.85 ± 18.96
	D (*n* = 8)^3^		33.59 ± 14.09	42.21 ± 14.64	36.34 ± 14.07
Left FT (milliseconds)	A (*n* = 10)		227.89 ± 38.70	215.51 ± 38.36	214.35 ± 31.16
	B (*n* = 11)		242.27 ± 79.14	242.53 ± 84.47	249.08 ± 78.58
	C (*n* = 10)		224.77 ± 43.78	206.18 ± 38.96	209.88 ± 18.94
	D (*n* = 8)^4^		211.50 ± 30.03	201.15 ± 34.00	205.03 ± 44.19
Right FT (milliseconds)	A (*n* = 10)		222.88 ± 52.50	210.66 ± 45.32	212.84 ± 48.05
	B (*n* = 11)		236.95 ± 98.00	235.03 ± 89.26	232.60 ± 72.61
	C (*n* = 10)		240.38 ± 72.60	191.64 ± 31.21	199.78 ± 33.62
	D (*n* = 8)^4^		189.58 ± 17.50	183.89 ± 20.82	181.63 ± 22.47
HDRS (points)	A (*n* = 10)		6.30 ± 6.73	-	5.70 ± 7.33
	B (*n* = 11)		4.64 ± 3.91		2.09 ± 2.21
	C (*n* = 10)		5.20 ± 5.59		1.30 ± 2.00
	D (*n* = 9)		3.78 ± 2.77		2.33 ± 3.20
PDQ-39 (points)	A (*n* = 10)		22.24 ± 19.93	-	19.87 ± 21.16
	B (*n* = 11)		26.44 ± 14.23		23.62 ± 12.09
	C (*n* = 10)		22.46 ± 11.12		18.48 ± 11.26
	D (*n* = 9)		18.44 ± 8.82		21.66 ± 9.65
Left Hemisphere CSP (miliseconds)	A (*n* = 8)	128.4 ± 49.2		151.8 ± 63.6	148.0 ± 33.8
	B (*n* = 10)	128.8 ±. 29.7		128.9 ± 43.6	125.0 ± 43.6
	C (*n* = 10)	129.8 ± 26.5		168.7 ± 27.7	148.9 ± 24.4
	D (*n* = 8)^4^	135.7 ± 33.6		132.0 ± 30.5	137.6 ± 38.0
Right Hemisphere CSP (miliseconds)	A (*n* = 8)	144.8 ± 58.5		167.7 ± 52.3	159.3 ± 53.5
	B (*n* = 10)	107.1 ± 24.3		123.3 ± 48.3	121.4 ± 38.9
	C (*n* = 10)	138.0 ± 29.9		176.7 ± 39.4	150.0 ± 40.2
	D (*n* = 8)^4^	138.3 ± 42.2		142.9 ± 35.0	141.4 ± 49.2

^1^Group A: rTMS; Group B: EEG-guided NFB protocol; Group C: combined (rTMS + EEG-guided NFB), Group D: no intervention. Results are shown as mean ± SD

^2^In Group B, data at all end-points are shown for *N* = 10 participants because of technical issues while collecting data from 1 participant

^3^In Group D, data at T0 are shown for *N* = 8 participants because of technical issues while collecting data from 1 participant

^4^In Group D, data at all end-points are shown for *N* = 8 participants because of technical issues while collecting data from 1 participant

*Abbreviations* CSP = Cortical Silent Period; FT = Finger Tapping; HDRS = Hamilton Depression Rating Scale; LOS = Limits of Stability; PDQ-39 = Parkinson´s Disease Questionnaire; TUG = Timed Up and Go Test; UPDRS-III = Unified Parkinson’s Disease Rating Scale-Part III, motor examination

**Fig. 2:**
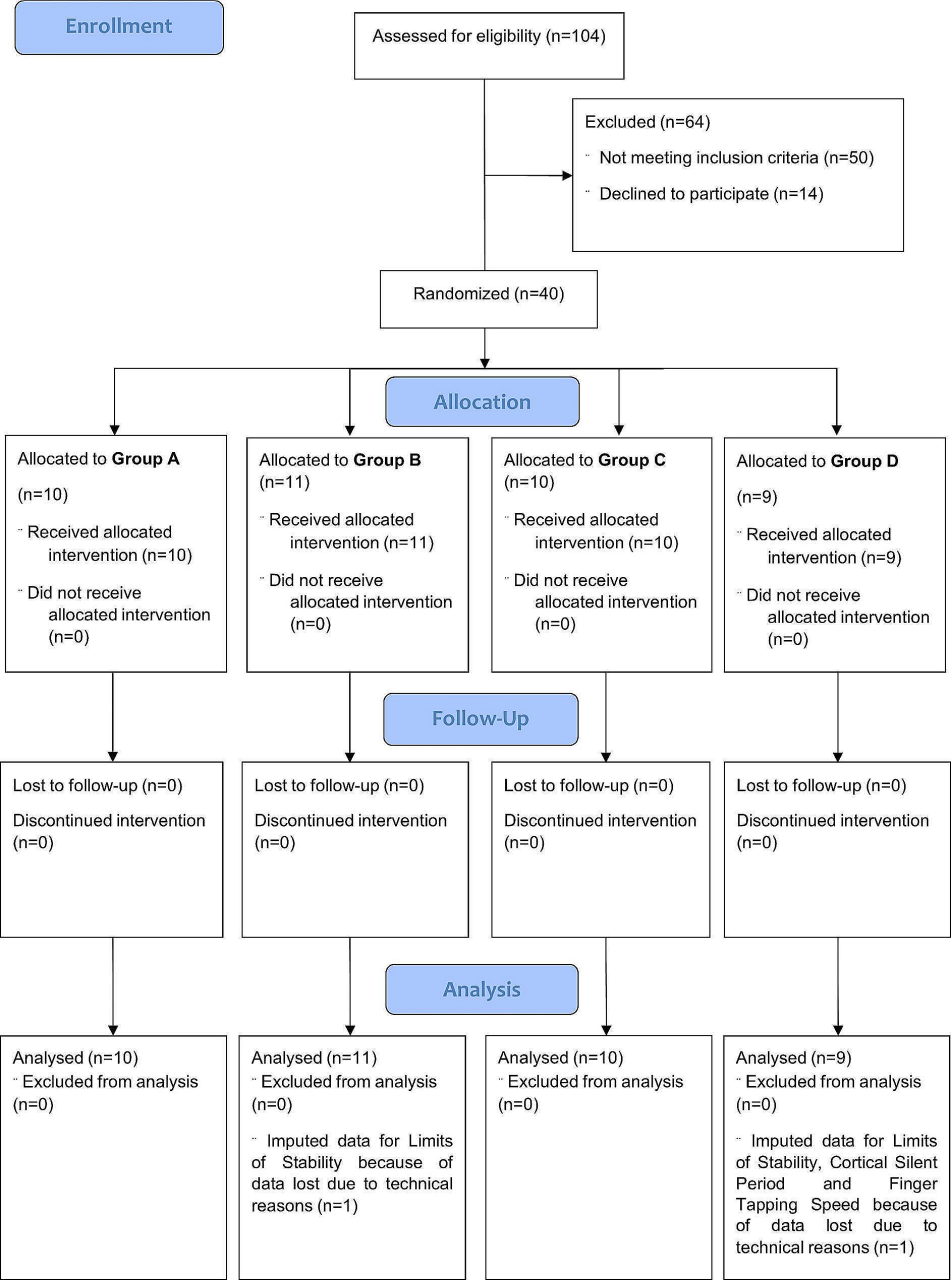
CONSORT flow diagram showing participant flow through each stage of the trial

All participants completed all the intervention sessions and assessments (Fig. 2), between January 2017 and May 2019. However, neurophysiological data were lost from one participant (Group D) at all end-points, postural stability data were lost from two participants (Group D at baselines and group B at all end-points) and motor speed data were lost from one participant (Group D) at all end-points, all because of technical issues during assessment. Therefore, missing data was present in *n* = 22 observations (1.45% of the overall data analysed).

### Primary outcome measure: motor symptoms severity (UPDRS-III)

**Fig. 3:**
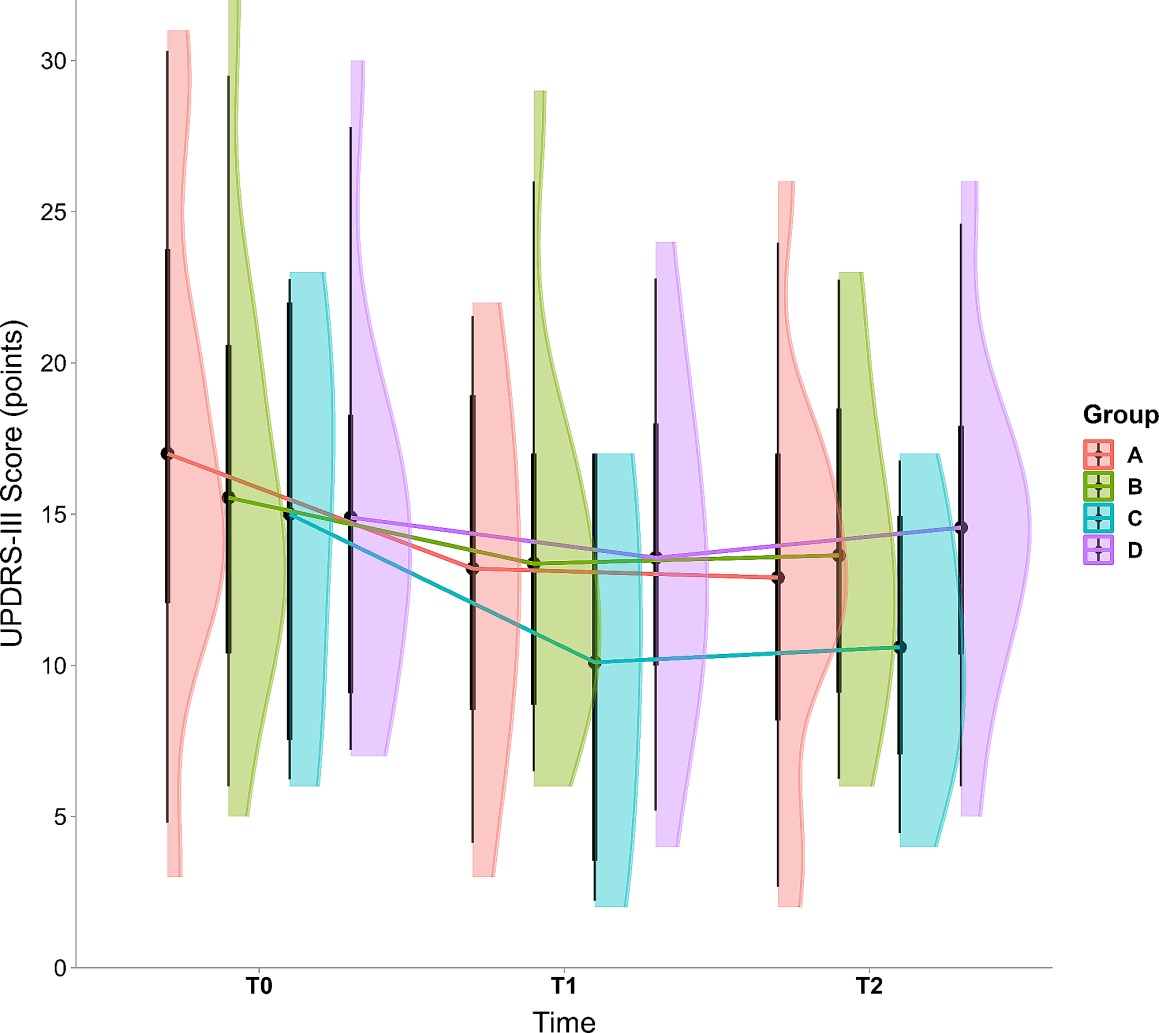
Changes in the Unified Parkinson’s Disease Rating Scale-III (UPDRS-III) from baseline (T0) to post-intervention (T1) and follow-up (T2). Group A received bilateral, high-frequency Repetitive Transcranial Magnetic Stimulation (rTMS). Group B received EEG-guided neurofeedback (EEG-guided neurofeedback). Group C received a combination of rTMS and EEG-guided neurofeedback. Group D did not receive any intervention. Points represent means, thick lines represent standard errors and thin lines represent range

The concordance between the two neurologists for rating the video-recorded UPDRS-III was high (kappa = 0.72). Regarding the overall Average Treatment Effect (ATE) on the UPDRS-III (Fig. 3), Group C showed the largest effect (ATE = -3.78 points, 95% CI [-6.05, -1.5], t_(35)_ = -3.125, *p* = 0.004), followed by Group A (ATE = -2.43 points [-4.71, -0.14], t_(35)_ = -1.99, *p* = 0.054). Negligible effects were observed in Group B (ATE = -0.1 points [-3.22, 1.22], t_(35)_ = -0.84, *p* = 0.41).

A group-by-time interaction was not found (F_(6, 35.49)_ = 2.01, *p* = 0.09). At T1 and T2 separately, Group C showed the largest ATE, always followed by Group A and B (Table 3).

**Table 3:** Estimates of the average treatment effects (ATE) according to analysis of covariance (ANCOVA) mixed-effects models at post-intervention (T1) and at follow-up (T2)

Variable		Group A					Group B					Group C				
		ATE	95% CI	P-Value	d	95% CI	ATE	95% CI	P-Value	d	95% CI	ATE	95% CI	P-Value	d	95% CI
UPDRS-III (points)	**T1**	-1.78	-4.75–1.20	0.237	-0.06	-0.96–0.84	-0.63	-3.53–2.27	0.664	-0.03	-0.91–0.85	-3.53	-6.49 – -0.57	**0.020**	-0.59	-1.5–0.34
	**T2**	-3.08	-6.05 – -0.10	**0.043**	-0.26	-1.16–0.65	-1.36	-4.26–1.54	0.352	-0.16	-1.04–0.72	-4.03	-6.99 – -1.07	**0.008**	-0.77	-1.7–0.18
TUG (seconds)	**T1**	0.93	-0.47–2.34	0.191	0.25	-0.66–1.15	-0.23	-1.61–1.15	0.741	-0.45	-1.33–0.45	0.23	-1.22–1.69	0.750	-0.49	-1.4–0.43
	**T2**	-0.32	-1.73–1.09	0.650	-0.35	-1.25–0.56	-0.51	-1.89–0.87	0.463	-0.51	-1.4–0.39	-0.11	-1.57–1.35	0.880	-0.56	-1.48–0.36
LOS (%)	**T1**	-1.33	-11.96–9.29	0.803	-0.39	-0.53–1.29	-1.08	-11.28–9.12	0.834	-0.07	-0.97–0.83	-5.48	-15.90–4.94	0.298	-0.31	-1.21–0.6
	**T2**	1.96	-8.70–12.62	0.715	0.63	-0.3–1.55	3.44	-6.76–13.64	0.504	0.32	-0.59–1.22	2.49	-7.93–12.91	0.636	0.20	-0.7–1.1
Left FT (ms)	**T1**	-4.46	-30.26–21.34	0.731	-0.39	-0.55–1.33	10.47	-15.02–35.96	0.415	0.61	-0.34–1.53	-11.18	-36.95–14.59	0.390	0.14	-0.8–1.07
	**T2**	-1.55	-27.34–24.24	0.905	-0.25	-0.69–1.18	21.09	-4.38–46.56	0.103	0.66	-0.28–1.59	-3.41	-29.18–22.35	0.792	0.15	-0.78–1.08
Right FT (ms)	**T1**	0.45	-23.61–24.50	0.971	0.73	-0.24–1.68	15.45	-8.34–39.25	0.199	0.73	-0.22–1.67	-30.22	-54.59 – -5.84	**0.016**	0.29	-0.65–1.22
	**T2**	10.09	-13.95–34.12	0.405	0.80	-0.18–1.76	20.49	-3.27–44.26	0.090	0.89	-0.08–1.83	-14.62	-38.97–9.73	0.235	0.62	-0.34–1.56
Left CSP (ms)	**T1**	22.0	-06.6–50.6	0.129	0.38	-0.56–1.32	-0.13	-2.92–2.67	0.928	-0.09	-0.97–0.79	3.79	0.93–6.64	**0.010**	1.27	0.22–2.28
	**T2**	14.3	-14.3–42.8	0.322	0.28	-0.66–1.21	-0.91	-3.70–1.88	0.519	-0.31	-1.22–0.61	1.41	-1.44–4.27	0.327	0.35	-0.59–1.29
Right CSP (ms)	**T1**	14.7	-17.9–47.4	0.371	0.54	-0.41–1.48	-0.30	-3.60–3.01	0.858	-0.45	-1.37–0.46	2.86	-0.40–6.12	0.085	0.90	-0.09–1.87
	**T2**	11.7	-21.0–44.3	0.479	0.35	-0.6–1.28	0.05	-3.25–3.35	0.975	-0.46	-1.38–0.47	0.72	-2.54–3.99	0.660	0.19	-0.74–1.12

^*^p < 0.05

*Abbreviations* CI: Confidence Interval; CSP = Cortical Silent Period; d = Cohen’s d; FT = Finger Tapping; HDRS = Hamilton Depression Rating Scale; LOS = Limits of Stability; PDQ-39 = Parkinson´s Disease Questionnaire; TUG = Timed Up and Go Test; UPDRS-III = Unified Parkinson’s Disease Rating Scale-Part III, motor examination

*Note* All ATE are shown in comparison to the control group (Group D) and in the same units as the corresponding variable

### Secondary outcome measures

#### Functional mobility and postural stability

For the overall ATE on TUG, negligible differences were found between Groups A-C and Group D (Group B: ATE = -0.37 s [-1.57, 0.83], t_(35)_ = -0.58, *p* = 0.57; Group A: ATE = 0.3 s [-0.92, 1.53], t_(35)_ = 0.47, *p* = 0.64; Group C: ATE = 0.06 s [-1.21, 1.34], t_(35)_ = 0.09, *p* = 0.93). A group-by-time interaction was not found (F_(6, 35.49)_ = 1.31, *p* = 0.28).

For the overall ATE on LOS, negligible differences were found between Groups A-C and Group D (Group C: ATE = -1.63% [-10.4, 7.14], t_(35)_ = -0.35, *p* = 0.73; Group A: ATE = 0.22% [-8.8, 9.24], t_(35.02)_ = 0.05, *p* = 0.96; Group B: ATE = 0.81% [-7.77, 9.39], t_(35)_ = 0.18, *p* = 0.86). A group-by-time interaction was not found (F_(6, 35.68)_ = 0.66, *p* = 0.68).

#### Motor speed

For the overall ATE on left FT, a slight decrease in tapping time was observed in Group C (ATE = -2.36 milliseconds [-23.48, 18.77], t_(35)_ = -0.21, *p* = 0.84), whereas an increase was found in Group B (ATE = 20.87 milliseconds [-0.13, 41.88], t_(35)_ = 1.87, *p* = 0.07) and Group A (ATE = 1.96 milliseconds [-19.2, 23.12], t_(35)_ = 0.17, *p* = 0.86). A group-by-time interaction was not found (F_(6, 35.5)_ = 1.05, *p* = 0.41).

For the overall ATE on right FT, a decrease in tapping time was only observed in Group C (ATE = -19.43 milliseconds [-39.09, 0.22], t_(35.06)_ = -1.86, *p* = 0.07). An increase was observed in Group B (ATE = 20.94 milliseconds [1.76, 40.1], t_(35.06)_ = 2.05, *p* = 0.05) and Group A (ATE = 8.13 milliseconds [-11.2, 27.46], t_(35.03)_ = 0.79, *p* = 0.43). A group-by-time interaction was found (F_(6, 35.52)_ = 3.24, *p* = 0.01), whereby an increase in tapping speed was present for Group C at T1 (Table 3).

#### Depression and health-related quality-of-life

In the HDRS at T2, Group A showed a slight increase in depression levels compared to Group D (ATE = 2.08 points [-1.19, 5.35], t_(35)_ = 1.29, *p* = 0.21, Cohen’s d = 0.58 [-0.35, 1.50]). A negligible effect was found in Group B (ATE = -0.68 points [-3.83, 2.47], t_(35)_ = -0.44, *p* = 0.66, Cohen’s d = -0.09 [-0.97, 0.79]) and a slight decrease in Group C (ATE = -1.76 points [-4.99, 1.48], t_(35)_ = -1.1, *p* = 0.28, Cohen’s d = -0.39 [-1.30, 0.52]).

In the PDQ-39 at T2, all groups showed improvements in health-related quality-of-life. Group C showed the largest effect (ATE = -6.89 points [-12.19, -1.61], t_(35)_ = -2.65, *p* = 0.01, Cohen’s d = -0.30 [-1.20, 0.61]), followed by group B (ATE = -5.43 [-10.69, -0.17], t_(35)_ = -2.1, *p* = 0.043, Cohen’s d = -0.18 [-0.71, 1.06]) and group A (ATE = -5.29 [-10.59, -0.01], t_(35)_ = -2.03, *p* = 0.05, Cohen’s d = -0.11 [-1.01, 0.80]).

#### Neurophysiological measures

For the overall ATE on left hemisphere CSP, Groups C and A showed an increase compared to Group D (Group C: ATE = 24.01 milliseconds [1.07, 46.95], t_(35)_ = 1.97, *p* = 0.057; Group A: ATE = 16.08 milliseconds [-6.87, 39.02], t_(35)_ = 1.32, *p* = 0.2), whereas Group B a slight decrease (ATE = -7.21 milliseconds [-29.65, 15.25], t_(35)_ = -0.6, *p* = 0.55). ). A group-by-time interaction was not found (F_(6, 35.49)_ = 1.98, *p* = 0.09).

For the overall ATE on right hemisphere CSP, Group C showed an increase compared to Group D (ATE = 12.32 milliseconds [-16.76, 41.41], t_(35)_ = 0.8, *p* = 0.43), whereas Groups A and B showed a slight decrease (Group A: ATE = -8.29 milliseconds [-20.96, 37.55], t_(35)_ = 0.53, *p* = 0.59; Group B: ATE = -9.92 milliseconds [-38.89, 19.05], t_(35)_ = -0.64, *p* = 0.52). ). A group-by-time interaction was not found (F_(6, 35.5)_ = 0.92, *p* = 0.5).

### Sensitivity analysis

Results from sensitivity analyses are shown in Supplementary Tables 5–9. For LOS, left FT and right CSP, point estimates (ATE) varied slightly across imputation methods, but uncertainty remained comparable as shown by similar 95% CI coverage, and statistical significance was unchanged.

For right FT, the overall ATE for Group C became statistically significant only for the unconditional mean imputation model, although the magnitude of the effect was similar to the other methods (ATE = -21.07 milliseconds [-41.92, -0.22]). For Group B, the ATE was statistically significant only for predictive mean matching and Bayesian linear regression, although the magnitude of the effect was similar to the other methods (ATE = 20.94 milliseconds [0.6, 41.28]). The remaining results were comparable across different imputation methods.

For left CSP, the overall ATE became statistically significant for the model with no imputed data, although the magnitude of the effect was similar to the other methods (ATE = 28.05 milliseconds [0.47–0.96]). The remaining results were equivalent across imputation methods.

## Discussion

Research into the efficacy of neuromodulation is still beginning to develop, particularly concerning its applications for PD. A significant gap in our knowledge exists regarding the synergistic potential of exogenous neuromodulation methods like rTMS and endogenous methods such as EEG-guided NFB, especially when these are used on patients with optimized pharmacological treatments. The principal objective of our investigation was to evaluate the impact of a combined regimen of non-invasive neuromodulation approaches on both the motor and non-motor symptoms of PD. Our findings indicate that the concomitant use of these neuromodulation techniques yielded a more pronounced amelioration of motor deficits and an enhancement in the health-related quality of life for patients, surpassing the outcomes achieved by each modality in isolation.

### Effects on motor symptoms

Previous meta-analyses suggest that high-frequency rTMS alone can improve motor symptoms in PD [48, 49]. This effect was replicated in our study (Group A), as in previous studies with similar designs [26].

Regarding the effects of NFB on motor symptoms severity, our results are consistent with recent work showing that fMRI-based NFB does not significantly improve them as measured by the UPDRS-III [17] and that it does not have additional effects to motor training [16]. The goal of our NFB protocol was achieving beta desynchronization (specifically, movement related beta decrease) [50] described as the bilateral reduction in beta power at the onset of movement [51]. Nevertheless, there is evidence from nonhuman primates research that such training could alleviate motor symptoms associated with PD compared to control conditions [52]. Additionally, studies indicate that this type of NFB might enhance postural stability in individuals with PD [53]. The lack of effect of our paradigm in terms of motor components may be justified by the fact that the reduction of bilateral alpha and beta power was not related to explicit instructions to perform any motor command, and this could therefore explain that the beta desynchronization may not have been related to sensorimotor network activation.

The combination of rTMS with EEG-guided NFB for ameliorating motor and non-motor symptoms of PD is a novel paradigm presented in this study. In line with our hypothesis, we observed larger effects of this combined protocol on overall motor symptoms, at specific time points and on average. However, specific components such as balance or postural stability were not improved to the same extent, likely reflecting that postural control is a complex function comprising different brain networks [54] that may not be sufficiently influenced by a focal stimulation of M1 with rTMS, whereas could be slightly more influenced by a more widespread neuromodulation as is shown by the tendency to improvement observed with NFB in TUG, or with combined rTMS + NFB in LOS. Considering tapping speed − a direct motor skill linked to bradykinesia in PD − only the combined intervention led to significant improvements in right FT. The left FT did not show significant changes for any treatment group. Considering that the effect is only evident in the dominant hand, and prior research has indicated that M1 activation via rTMS can enhance FT speed [55], our findings suggest that when rTMS alone does not suffice, the effect may be increased by NFB. In this line, data show that handedness is associated with differences in effective connectivity within the human motor network with a prominent role of the Supplementary Motor Area in right-handers, as 90% of our sample [56]. It can be hypothesized that NFB alone might not produce noticeable effects on the motor circuits, but it seems that its effect on the Supplementary Motor Area, that has been reported to be almost inevitable when targeting M1 [57], may potentiate the interaction with motor networks when applied after rTMS.

### Non-motor symptoms and quality-of-life

Although the effects of rTMS over the dorsolateral prefrontal cortex on mood symptoms in PD are well studied, generally showing improvement of depression [58, 59], we did not expect any direct effects of neuromodulation on HDRS [60], which has been confirmed by our results. Most of the slight although negligible results found for NFB and rTMS + NFB protocols may be influenced by the improvement found in other variables or the motivating aspect of the NFB experience, that may have a ludic component. Nevertheless, our results regarding the effects on depressive symptoms in PD should be considered cautiously as they are based on a sample with mild levels of depression or no depression, as it was not the main goal of our research. Future studies are needed to confirm these results, as there is recent evidence that EEG-guided NFB is useful in the management of major depression disorder [61, 62].

Nevertheless, positive results in PDQ-39 were observed across protocols. Importantly, all treatments produced improvements which were larger than the minimal clinically important difference (MCID: -4.72 points) [63], the combined intervention showing the largest effect, probably because the motor improvement was also more evident in this group. To our knowledge, this is the first time that non-invasive neuromodulation after-effects show improvements beyond purely physical or psychological domains in PD.

### Neurophysiological effects

In our study, we observed an elongation of CSP for rTMS and rTMS + NFB protocols compared to no intervention, and this effect was of similar magnitude in both. Nonetheless, no effect was observed for NFB. This suggests that the effects on CSP can be primarily associated to direct cortical stimulation through rTMS, producing an increase in intracortical inhibition mediated by gamma aminobutyric acid, which has been previously described as implying greater motor control [12]. Furthermore, our findings are consistent with studies showing that CSP duration can be prolonged in PD after either facilitatory (5, 25–50 Hz) [64–66] or inhibitory (1 Hz) rTMS over M1 [65, 67], an effect which has not been found after other stimulation modalities, for example intermittent theta-burst stimulation [68]. Although the physiological mechanisms underpinning these effects are poorly understood, some studies in healthy individuals suggest they could be mediated by temporal summation of inhibitory interneurons [69]. Interestingly, our results showed changes in CSP with rTMS, although did not clearly show an effect of NFB on intracortical inhibition. This indicates that there might be other plausible neurophysiological mechanisms underlying NFB, not directly related to cortical excitability, that justify some of the clinical outcomes of protocols including NFB.

The observed effects in our study likely result from the synergistic impacts of high-frequency rTMS and NFB techniques. Specifically, high-frequency rTMS targeting the M1 region appears to alter striatal activity by stimulating the motor cortex-thalamus-basal ganglia pathway. This stimulation may regulate inhibitory signals within the medial globus pallidus, positively affecting the motor cortex [70]. Furthermore, this intervention has been associated with the local release of endogenous dopamine in the ipsilateral putamen via cortico-basal ganglia pathways [71].

In relation to NFB, modifying alpha frequencies in motor areas has been shown to enhance corticospinal excitability [19], and significantly impacts dopamine release in the frontal cortex [72]. Although many underlying mechanisms remain unveiled, these mechanisms together may help to understand the clinical effects observed in our protocol.

### Limitations

The main limitation of this study was the small sample size of each arm, which decreased statistical precision and power. Additionally, allocation was performed in blocks based on the severity of motor symptoms. This has the advantage of making all groups comparable although a fully randomized assignment could have been desirable. The lack of sham conditions for both rTMS and NFB is also a substantial limitation. As we compared the effects of the protocols with no intervention, a placebo effect could not be completely ruled out. Our NFB paradigm was targeted for a reduction of the alpha and beta power in central electrodes, but specific motor tasks were not introduced to guarantee sensorimotor network activation. No calibration session was performed and accuracy and thresholds during each session was not recorded; these limitations may have influenced the effects of the NFB paradigm. The dosage of the interventions (8 sessions within 2 weeks), although equivalent to previous studies, may be considered short to produce large effect sizes on some of the outcome measures, like TUG or LOS. The participants were asked for the perceived difficulty for each scenario and no one found it difficult. A great majority of participants (< 90%) considered the duration of scenarios and number of sessions adequate, which makes feasible longer protocols without the interference of fatigue or motivation loss. The use of immersive VR might be a source of confounding variable. However, the immersive VR versus non-VR has been proven to produce EEG differences only in the theta and beta bands in the frontal midline (Fz channel) [73], which does not conflict with the targets of our study. Our post-intervention measurements only included an immediate assessment and short-term follow-up (15 days after treatment), and therefore the extent to which the observed effects remained beyond this period, is unknown. Further research with larger cohorts, longer treatment protocols and longer-term follow-ups is needed to confirm and extend these findings.

## Conclusions

This study suggests that combining bilateral high-frequency rTMS and EEG-guided NFB may have synergistic effects in improving motor symptoms and health-related quality-of-life in individuals with PD, but this effect may not be substantial regarding functional mobility, postural stability, motor speed or depressive symptoms. Nonetheless, these preliminary results offer valuable insights into the potential of combined non-invasive neuromodulation approaches, and they pave the way for future investigations and clinical applications in this field, with the goal of optimizing and personalizing neuromodulation therapies for individuals with PD.

### Electronic supplementary material

Below is the link to the electronic supplementary material.

Supplementary Material 1

## Data Availability

Scripts and data are freely accessible on the Open Science Framework at https://osf.io/54gmk/.
