# Supplementary material for: Clinical and neurophysiological effects of bilateral repetitive transcranial magnetic stimulation and EEG-guided neurofeedback in Parkinson’s disease: a randomized, four-arm controlled trial
Source: J Neuroeng Rehabil. 2024 Aug 5;21:135. doi: 10.1186/s12984-024-01427-5 (PMC11299373; doi:10.1186/s12984-024-01427-5)
Supplement: Supplementary file 1 — Supplementary Material 1 [file 12984_2024_1427_MOESM1_ESM.docx]

**Supplementary Materials**

**R packages used for statistical analysis**

Data wrangling was performed using the ‘tidyverse’ library [1] and data visualization with the ‘ggdist’ package [2]. ANCOVA models were created using the ‘lme4’ package [3]. The ATE was obtained as the overall effect across time using the formula ‘y ~ β_0_ + β_1_*treatment* + β_2_*subject* + β_3_*baseline*’, and at T1 and T2 separately using the formula ‘y ~ β_0_ + β_1_*treatment* + β_2_*subject* + β_3_*baseline* + β_4_*time +* β_5_*treatment* x *time*’, as detailed in Twisk et al [4]. For the HDRS and PDQ39, which were only administered at follow-up, the ANCOVA model could not include random effects nor treatment interactions with time, and therefore they were modelled as ‘y ~ β_0_ + β_1_*treatment* + β_2_*baseline*’. Therefore, all models accounted for baseline measures, as covariates. Effect sizes were obtained with the ‘effectsize’ package [5]. Missing data were imputed using the ‘mice’ package [6].

1. Wickham H, Averick M, Bryan J, Chang W, McGowan LD, François R, et al. Welcome to the Tidyverse. J Open Source Softw. 2019;4:1686.

2. Kay M. ggdist: Visualizations of distributions and uncertainty [Internet]. [cited 2023 Nov 14]. Available from: https://zenodo.org/records/7933524

3. Bates D, Mächler M, Bolker B, Walker S. Fitting Linear Mixed-Effects Models Using lme4. J Stat Softw. 2015;67:1–48.

4. Twisk J, Bosman B, Hoekstra T, Rijnhart J, Welten M, Heymans M. Different ways to estimate treatment effects in randomised controlled trials. Contemp Clin Trials Commun. 2018;10:80–5.

5. Ben-Shachar MS, Lüdecke D, Makowski D. effectsize: Estimation of Effect Size Indices and Standardized Parameters. J Open Source Softw. 2020;5:2815.

6. Buuren S van, Groothuis-Oudshoorn K. mice: Multivariate Imputation by Chained Equations in R. J Stat Softw. 2011;45:1–67.

**Supplementary Tables 1a-1c.** Fixed and random effects aside from treatment effects, for the longitudinal analysis of covariance mixed-effects models, considering the overall treatment effect (modelled as ‘y ~ β_0_ + β_1_*treatment* + β_2_*subject* + β_3_*baseline*’).

In all Tables: σ^2^ = mean random effect variance. τ_00 =_ random intercept variance. ICC = Intraclass Correlation Coefficient. Marginal R^2^ = variance of the fixed effects. Conditional R^2^ = variance of the fixed and random effects. CI = Confidence Interval. p = p-value.

**Supplementary Table 1a.** Unified Parkinson’s disease Rating Scale (UPDRS), Timed Up and Go Test (TUG) and Limits of Stability (LOS).

|  | **UPDRS** | | | **TUG** | | | **LOS** | | |
| --- | --- | --- | --- | --- | --- | --- | --- | --- | --- |
| *Predictors* | *Estimates* | *95% CI* | *p* | *Estimates* | *95% CI* | *p* | *Estimates* | *95% CI* | *p* |
| (Intercept) | 4.03 | 1.52 – 6.55 | **0.002** | 2.02 | -0.62 – 4.66 | 0.131 | 14.34 | 4.83 – 23.86 | **0.004** |
| Baseline | 0.67 | 0.55 – 0.79 | **<0.001** | 0.76 | 0.54 – 0.97 | **<0.001** | 0.71 | 0.50 – 0.92 | **<0.001** |
| **Random Effects** | |  |  |  |  |  |  |  |  |
| σ^2^ | 6.50 | | | 0.70 | | | 52.45 | | |
| τ_00_ | 3.68 _Subject_ | | | 1.66 _Subject_ | | | 77.06 _Subject_ | | |
| ICC | 0.36 | | | 0.70 | | | 0.50 | | |
| N | 40 _Subject_ | | | 40 _Subject_ | | | 40 _Subject_ | | |
| Observations | 80 | | | 80 | | | 80 | | |
| Marginal R^2^ / Conditional R^2^ | 0.698 / 0.807 | | | 0.545 / 0.865 | | | 0.501 / 0.798 | | |

**Supplementary Table 1b.** Left and right Finger Tapping Speed (FT).

|  | **Left FT** | | | **Right FT** | | |
| --- | --- | --- | --- | --- | --- | --- |
| *Predictors* | *Estimates* | *95% CI* | *p* | *Estimates* | *95% CI* | *p* |
| (Intercept) | 23.51 | -13.18 – 60.19 | 0.206 | 56.79 | 31.47 – 82.11 | **<0.001** |
| Baseline | 0.83 | 0.68 – 0.98 | **<0.001** | 0.66 | 0.55 – 0.76 | **<0.001** |
| **Random Effects** | | | | | | |
| σ^2^ | 347.54 | | | 317.75 | | |
| τ_00_ | 422.10 _Subject_ | | | 332.16 _Subject_ | | |
| ICC | 0.55 | | | 0.51 | | |
| N | 40 _Subject_ | | | 40 _Subject_ | | |
| Observations | 80 | | | 80 | | |
| Marginal R^2^ / Conditional R^2^ | 0.728 / 0.877 | | | 0.776 / 0.890 | | |

**Supplementary Table 1c.** Left and right hemisphere Cortical Silent Periods (CSP).

|  | **Left CSP** | | | **Right CSP** | | |
| --- | --- | --- | --- | --- | --- | --- |
| *Predictors* | *Estimates* | *95% CI* | *p* | *Estimates* | *95% CI* | *p* |
| (Intercept) | 47.32 | 11.70 – 82.95 | **0.010** | 67.35 | 25.92 – 108.78 | **0.002** |
| Baseline | 0.67 | 0.43 – 0.91 | **<0.001** | 0.61 | 0.34 – 0.87 | **<0.001** |
| **Random Effects** | | | | | | |
| σ^2^ | 562.17 | | | 638.18 | | |
| τ_00_ | 425.51 _Subject_ | | | 811.26 _Subject_ | | |
| ICC | 0.43 | | | 0.56 | | |
| N | 40 _Subject_ | | | 40 _Subject_ | | |
| Observations | 80 | | | 80 | | |
| Marginal R^2^ / Conditional R^2^ | 0.423 / 0.671 | | | 0.379 / 0.727 | | |

**Supplementary Tables 2a-2d.** Fixed and random effects aside from treatment effects, for the longitudinal analysis of covariance mixed-effects models, considering the treatment effects at post-intervention and follow-up separately (modelled as ‘y ~ β_0_ + β_1_*treatment* + β_2_*subject* + β_3_*baseline* + β_4_*time +* β_5_*treatment* x *time*’).

In Tables 2a, 2b and 2d: σ^2^ = mean random effect variance. τ_00 =_ random intercept variance. ICC = Intraclass Correlation Coefficient. Marginal R^2^ = variance of the fixed effects. Conditional R^2^ = variance of the fixed and random effects. CI = Confidence Interval. p = p-value.

**Supplementary Table 2a.** Unified Parkinson’s disease Rating Scale (UPDRS), Timed Up and Go Test (TUG) and Limits of Stability (LOS).

|  | **UPDRS** | | | **TUG** | | | **LOS** | | |
| --- | --- | --- | --- | --- | --- | --- | --- | --- | --- |
| *Predictors* | *Estimates* | *CI* | *p* | *Estimates* | *CI* | *p* | *Estimates* | *CI* | *p* |
| (Intercept) | 3.53 | 0.73 – 6.34 | **0.014** | 1.84 | -0.83 – 4.51 | 0.173 | 16.93 | 6.85 – 27.02 | **0.001** |
| Time [1] | 1.00 | -1.49 – 3.49 | 0.427 | 0.36 | -0.40 – 1.12 | 0.348 | -5.35 | -12.18 – 1.47 | 0.122 |
| Baseline | 0.67 | 0.55 – 0.79 | **<0.001** | 0.76 | 0.54 – 0.97 | **<0.001** | 0.71 | 0.50 – 0.92 | **<0.001** |
| **Random Effects** | | | | | | | | | |
| σ^2^ | 7.04 | | | | 0.65 | | 2.73 | | |
| τ_00_ | 3.41 _Subject_ | | | | 1.69 _Subject_ | | 76.91 _Subject_ | | |
| ICC | 0.33 | | | | 0.72 | | 0.59 | | |
| N | 40 _Subject_ | | | | 40 _Subject_ | | 40 _Subject_ | | |
| Observations | 80 | | | | 80 | | 80 | | |
| Marginal R^2^ / Conditional R^2^ | 0.694 / 0.794 | | | | 0.553 / 0.875 | | 0.507 / 0.799 | | |

**Supplementary Table 2b.** Left and right Finger Tapping Speed (FT).

|  | **Left FT** | | | **Right FT** | | |
| --- | --- | --- | --- | --- | --- | --- |
| *Predictors* | *Estimates* | *95% CI* | *p* | *Estimates* | *95% CI* | *p* |
| (Intercept) | 21.11 | -16.81 – 59.04 | 0.271 | 58.48 | 31.91 – 85.04 | **<0.001** |
| Time [1] | 4.57 | -13.64 – 22.78 | 0.619 | -3.80 | -21.16 – 13.57 | 0.664 |
| Baseline | 0.83 | 0.68 – 0.99 | **<0.001** | 0.66 | 0.55 – 0.77 | **<0.001** |
| **Random Effects** | | | | | | |
| σ^2^ | 374.90 | | | 340.53 | | |
| τ_00_ | 408.42 _Subject_ | | | 320.77 _Subject_ | | |
| ICC | 0.52 | | | 0.49 | | |
| N | 40 _Subject_ | | | 40 _Subject_ | | |
| Observations | 80 | | | 80 | | |
| Marginal R^2^ / Conditional R^2^ | 0.725 / 0.868 | | | 0.774 / 0.883 | | |

**Supplementary Table 2c.** Hamilton Depression Rating Scale (HDRS) and Parkinson’s Disease Questionnaire-39 (PDQ-39). R^2^ = proportion of variance of the dependent variable explained by the independent variables. R^2^ adjusted = proportion of variance of the dependent variable explained by the independent variables, after adjuster for the number of independent variables. CI = Confidence Interval. p = p-value.

|  | **HDRS** | | | **PDQ-39** | | |
| --- | --- | --- | --- | --- | --- | --- |
| *Predictors* | *Estimates* | *95% CI* | *p* | *Estimates* | *95% CI* | *p* |
| (Intercept) | 0.57 | -2.69 – 3.83 | 0.725 | 4.62 | 0.07 – 9.16 | **0.047** |
| Baseline | 0.47 | 0.16 – 0.77 | **0.004** | 0.92 | 0.79 – 1.06 | **<0.001** |
| Observations | 40 | | | 40 | | |
| R^2^ / R^2^ adjusted | 0.484 / 0.425 | | | 0.852 / 0.835 | | |

**Supplementary Table 2d.** Left and right hemisphere Cortical Silent Periods (CSP).

|  | **Left CSP** | | | **Right CSP** | | |
| --- | --- | --- | --- | --- | --- | --- |
| *Predictors* | *Estimates* | *95% CI* | *p* | *Estimates* | *95% CI* | *p* |
| (Intercept) | 44.40 | 7.02 – 81.79 | **0.021** | 70.24 | 27.19 – 113.29 | **0.002** |
| Time [1] | 5.81 | -16.48 – 28.09 | 0.605 | -5.77 | -28.72 – 17.18 | 0.618 |
| Baseline | 0.67 | 0.43 – 0.91 | **<0.001** | 0.61 | 0.34 – 0.87 | **<0.001** |
| **Random Effects** | | | | | | |
| σ^2^ | 561.51 | | | 595.57 | | |
| τ_00_ | 425.84 _Subject_ | | | 832.56 _Subject_ | | |
| ICC | 0.43 | | | 0.58 | | |
| N | 40 _Subject_ | | | 40 _Subject_ | | |
| Observations | 80 | | | 80 | | |
| Marginal R^2^ / Conditional R^2^ | 0.432 / 0.677 | | | 0.396 / 0.748 | | |

**Supplementary Table 3.** ANOVA table of main effects on the dependent variables, considering the overall treatment effects across time (modelled as ‘y ~ β_0_ + β_1_*treatment* + β_2_*subject* + β_3_*baseline*’).

| Dependent variable | Regressor | DoF | F-statistic | P-Value |
| --- | --- | --- | --- | --- |
| UPDRS | Treatment | 3, 35 | 3.815 | **0.018*** |
|  | Baseline | 1, 35 | 122.867 | **5.46^-13*^** |
| TUG | Treatment | 3, 35 | 0.410 | 0.747 |
|  | Baseline | 1, 35 | 49.54 | **3.407^-8*^** |
| LOS | Treatment | 3, 35.01 | 0.108 | 0.955 |
|  | Baseline | 1, 35.34 | 45.01 | **8.636^-8*^** |
| Left FT | Treatment | 3, 35 | 1.961 | 0.14 |
|  | Baseline | 1, 35.09 | 116.38 | **1.087^-12*^** |
| Right FT | Treatment | 3, 35.03 | 6.05 | **0.002*** |
|  | Baseline | 1, 35.92 | 154.45 | **1.435^-14*^** |
| Left CSP | Treatment | 3, 35 | 2.987 | **0.044*** |
|  | Baseline | 1, 35 | 31.195 | **2.72^-6*^** |
| Right CSP | Treatment | 3, 35 | 0.805 | 0.499 |
|  | Baseline | 1, 35 | 20.29 | **7.092^-5*^** |

***P < 0.05.** Abbreviations: CSP = Cortical Silent Period; DoF: Degrees of Freedom; FT = Finger Tapping Speed; HDRS = Hamilton Depression Rating Scale; LOS = Limits of Stability; PDQ-39 = Parkinson´s Disease Questionnaire; TUG = Timed Up and Go Test; UPDRS-III = Unified Parkinson’s Disease Rating Scale-Part III, motor examination.

**Supplementary Table 4.** ANOVA table of main effects on the dependent variables, considering the treatment effects at post-intervention and follow-up separately (modelled as ‘y ~ β_0_ + β_1_*treatment* + β_2_*subject* + β_3_*baseline* + β_4_*time +* β_5_*treatment* x *time*’).

| Dependent variable | Regressor | DoF | F-statistic | P-Value |
| --- | --- | --- | --- | --- |
| UPDRS | Time | 1, 36 | 0.64 | 0.43 |
|  | Baseline | 1, 35 | 122.87 | **5.46^-13*^** |
|  | Treatment*Time | 6, 35.49 | 2.01 | 0.09 |
| TUG | Time | 1, 36 | 0.89 | 0.35 |
|  | Baseline | 1, 35 | 49.54 | **3.41^-8*^** |
|  | Treatment*Time | 6, 35.49 | 1.31 | 0.28 |
| LOS | Time | 1, 36.01 | 2.45 | 0.13 |
|  | Baseline | 1, 35.34 | 45.34 | **8.06^-13*^** |
|  | Treatment*Time | 6, 35.68 | 0.66 | 0.68 |
| Left FT | Time | 1, 36.01 | 0.25 | 0.62 |
|  | Baseline | 1, 35.08 | 116.5 | **1.08^-12*^** |
|  | Treatment*Time | 6, 35.5 | 1.05 | 0.41 |
| Right FT | Time | 1, 36.09 | 0.19 | 0.66 |
|  | Baseline | 1, 35.76 | 154.63 | **1.51^-14*^** |
|  | Treatment*Time | 6, 35.52 | 3.24 | **0.01*** |
| HDRS | Treatment | 3 | 3.192 | **0.04*** |
|  | Baseline | 1 | 19.7 | **8.65^-5*^** |
| PDQ-39 | Treatment | 3 | 1.63 | 0.2 |
|  | Baseline | 1 | 196.71 | 6.24^-16^ |
| Left CSP | Time | 1, 36 | 0.27 | 0.6 |
|  | Baseline | 1, 35 | 31.21 | **2.71^-6*^** |
|  | Treatment*Time | 6, 35.49 | 1.98 | 0.09 |
| Right CSP | Time | 1, 36 | 0.25 | 0.62 |
|  | Baseline | 1, 35 | 20.29 | **7.10^-5*^** |
|  | Treatment*Time | 6, 35.5 | 0.92 | 0.5 |

***P < 0.05.** Abbreviations: CSP = Cortical Silent Period; DoF: Degrees of Freedom; FT = Finger Tapping Speed; HDRS = Hamilton Depression Rating Scale; LOS = Limits of Stability; PDQ-39 = Parkinson´s Disease Questionnaire; TUG = Timed Up and Go Test; UPDRS-III = Unified Parkinson’s Disease Rating Scale-Part III, motor examination.

**Supplementary Figures 1-4.** Changes in secondary outcome measures from baseline (T0) to post-intervention (T1) and follow-up (T2). Points represent means, thick lines represent standard errors and thin lines represent range.


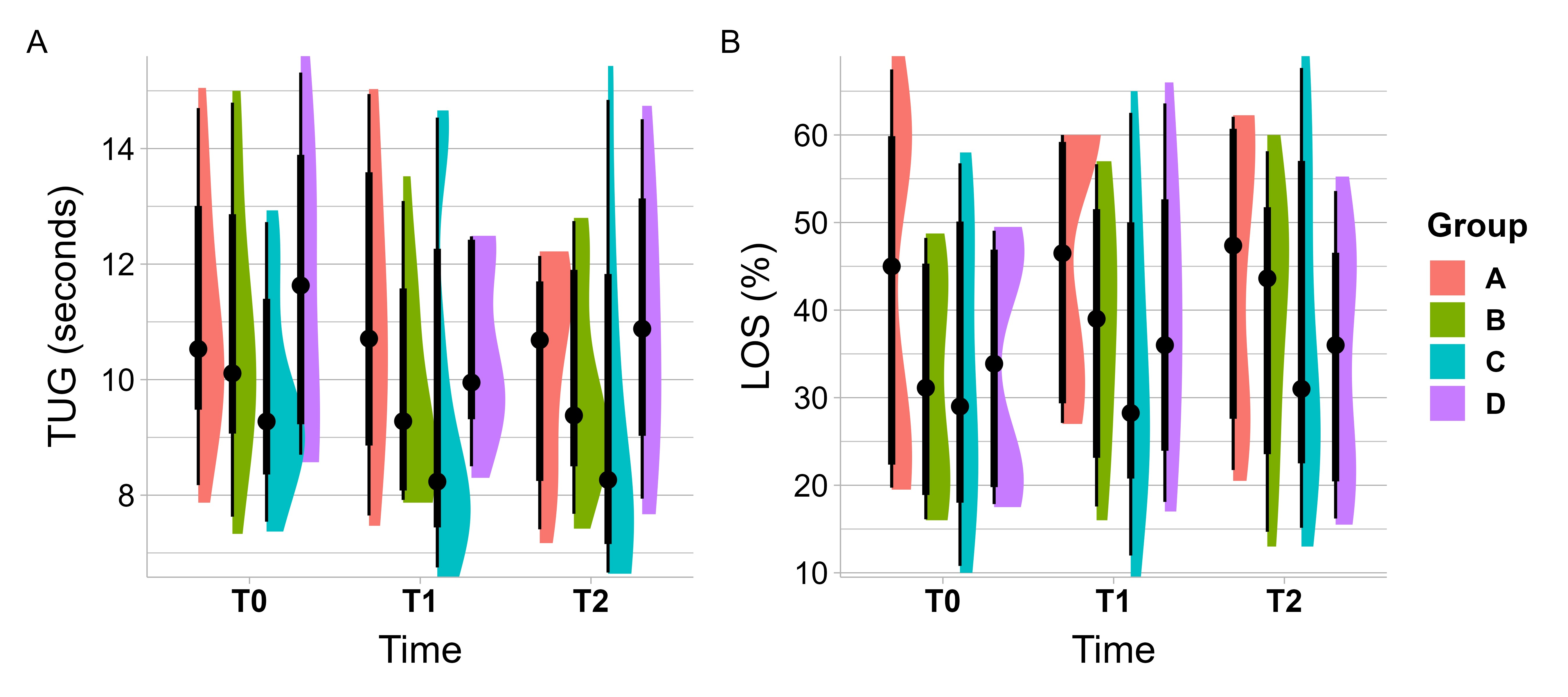


**Supplementary Figure 1.** Changes in the Timed Up and Go Test (TUG) and Limits of Stability (LOS).


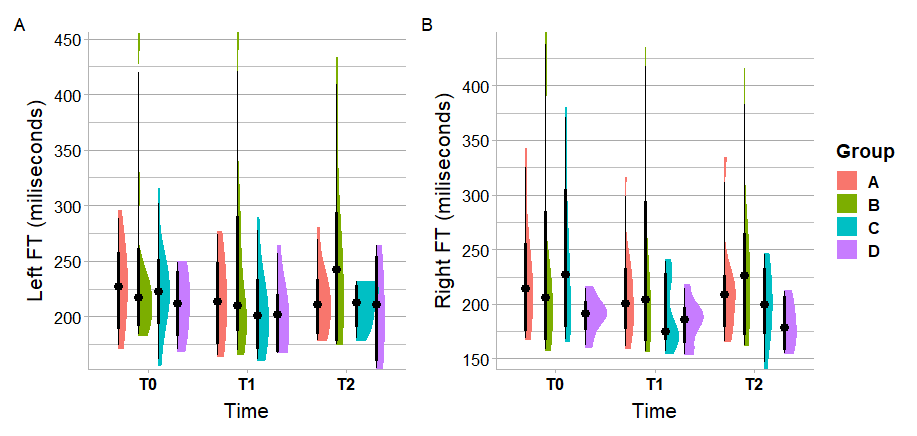


**Supplementary Figure 2.** Changes in left and right Finger Tapping Speed (FT).


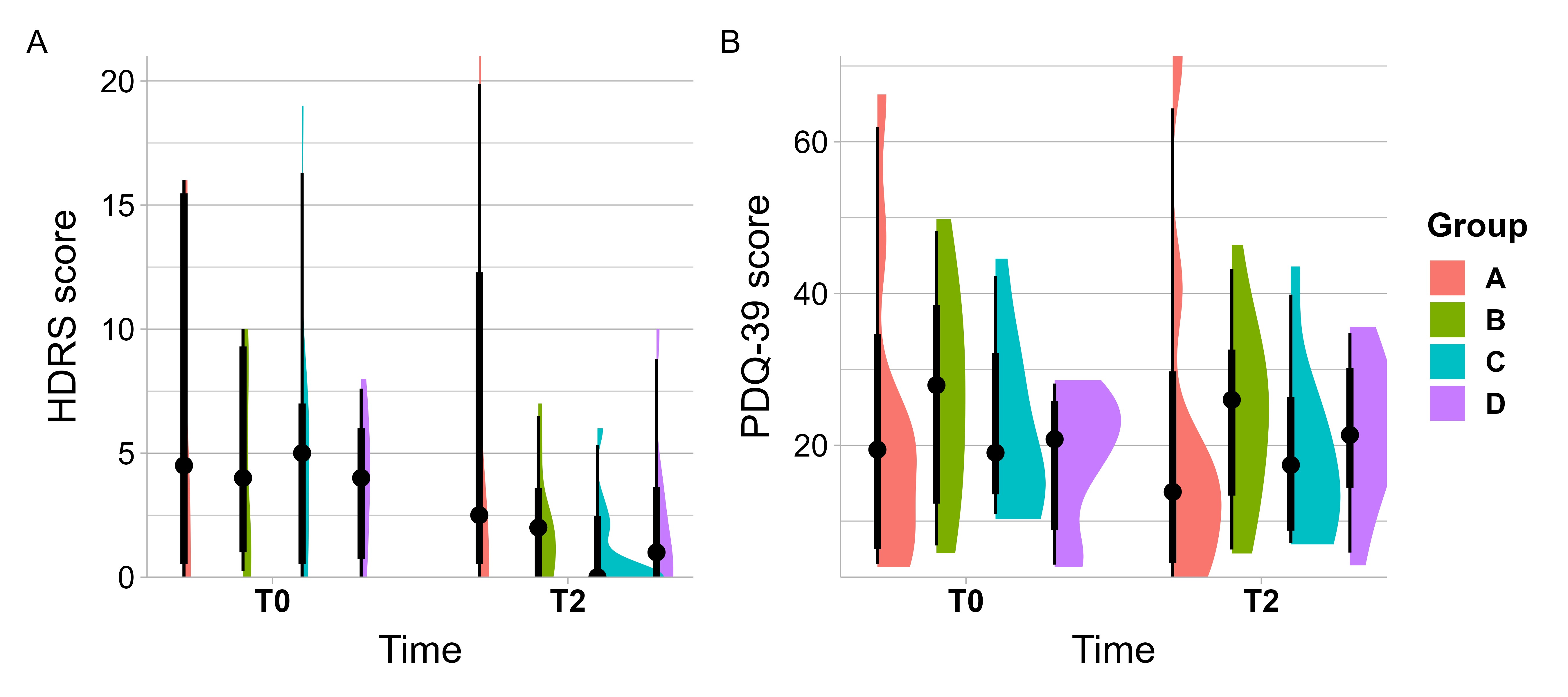


**Supplementary Figure 3.** Changes in Hamilton Depression Rating Scale (HDRS) and Parkinson’s Disease Questionnaire-39 (PDQ-39).


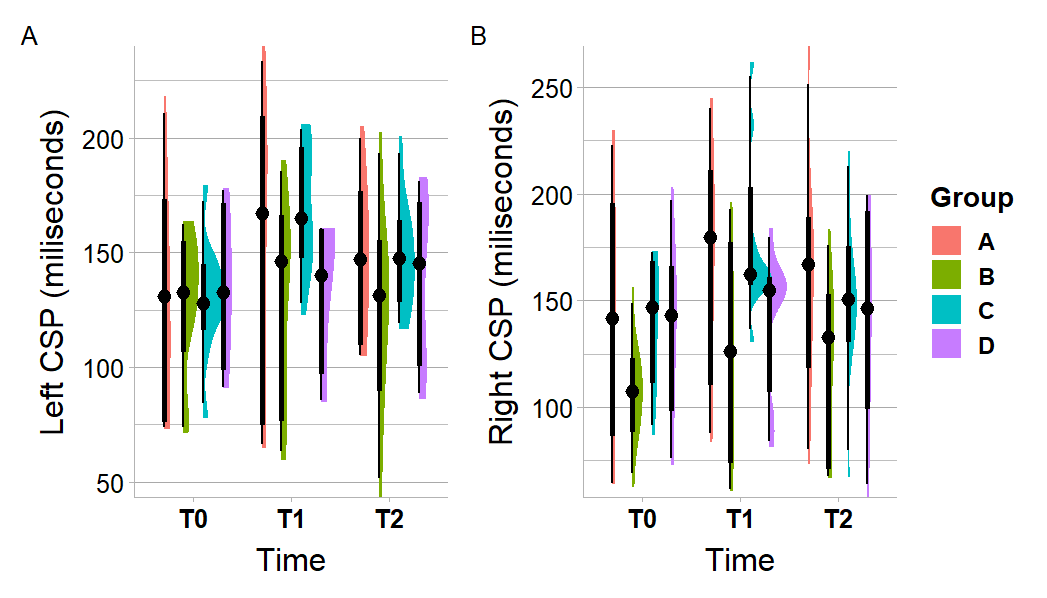


**Supplementary Figure 4.** Changes in left and right hemisphere Cortical Silent Period (CSP).

**Sensitivity analysis for missing data procedures**

We performed sensitivity analyses considering other imputation methods aside from Predictive Mean Matching (PMM), such as Random sampling from observed values, Unconditional mean imputation and Bayesian linear regression, or no imputation (i.e. case-wise deletion). The results are shown in the following tables.

**Supplementary Table 5.** Sensitivity analysis for Limits of Stability (LOS).

|  | **No imputation** | | | | **PMM** | | | | **Random sample** | | | **Unconditional mean imputation** | | | | **Bayesian Linear Regression** | | | |
| --- | --- | --- | --- | --- | --- | --- | --- | --- | --- | --- | --- | --- | --- | --- | --- | --- | --- | --- | --- |
| *Predictors* | *Estimates* | *CI* | *p* | *Estimates* | | *CI* | *p* | *Estimates* | | *CI* | *p* | | *Estimates* | *CI* | *p* | | *Estimates* | *CI* | *p* |
| (Intercept) | 16.39 | 6.31 – 26.47 | **0.002** | 14.34 | | 4.83 – 23.86 | **0.004** | 17.63 | | 7.23 – 28.02 | **0.001** | | 14.05 | 3.90 – 24.20 | **0.007** | | 16.93 | 8.10 – 25.77 | **<0.001** |
| Group [A] | -0.75 | -10.47 – 8.98 | 0.879 | 0.22 | | -9.35 – 9.80 | 0.963 | 3.08 | | -7.23 – 13.39 | 0.553 | | 1.79 | -7.95 – 11.53 | 0.715 | | 0.20 | -9.08 – 9.48 | 0.966 |
| Group [B] | 0.76 | -8.78 – 10.30 | 0.874 | 0.81 | | -8.30 – 9.91 | 0.861 | 1.65 | | -8.29 – 11.58 | 0.742 | | 3.06 | -6.28 – 12.40 | 0.516 | | 1.20 | -7.62 – 10.03 | 0.786 |
| Group [C] | -2.84 | -12.38 – 6.69 | 0.554 | -1.63 | | -10.94 – 7.68 | 0.728 | -0.15 | | -10.31 – 10.00 | 0.976 | | -0.35 | -9.89 – 9.19 | 0.942 | | -2.23 | -11.25 – 6.78 | 0.623 |
| LOS pre | 0.68 | 0.47 – 0.89 | **<0.001** | 0.71 | | 0.50 – 0.92 | **<0.001** | 0.56 | | 0.35 – 0.78 | **<0.001** | | 0.68 | 0.46 – 0.90 | **<0.001** | | 0.65 | 0.45 – 0.84 | **<0.001** |
| **Random Effects** | | | | | | | | | | | | | | | | | | | |
| σ^2^ | 53.48 | | | | 52.45 | | | | 62.99 | | | 50.86 | | | | 56.94 | | | |
| τ_00_ | 74.71 _Subject_ | | | | 77.06 _Subject_ | | | | 91.45 _Subject_ | | | 83.14 _Subject_ | | | | 68.25 _Subject_ | | | |
| ICC | 0.58 | | | | 0.59 | | | | 0.59 | | | 0.62 | | | | 0.55 | | | |
| N | 38 _Subject_ | | | | 40 _Subject_ | | | | 40 _Subject_ | | | 40 _Subject_ | | | | 40 _Subject_ | | | |
| Observations | 76 | | | | 80 | | | | 80 | | | 80 | | | | 80 | | | |
| Marginal R^2^ / Conditional R^2^ | 0.485 / 0.785 | | | | 0.501 / 0.798 | | | | 0.366 / 0.741 | | | 0.462 / 0.796 | | | | 0.486 / 0.766 | | | |

**Supplementary Table 6.** Sensitivity analysis for left Finger Tapping (FT) Speed.

|  | **No imputation** | | | | **PMM** | | | | **Random sample** | | | | **Unconditional mean imputation** | | | | **Bayesian Linear Regression** | |  |
| --- | --- | --- | --- | --- | --- | --- | --- | --- | --- | --- | --- | --- | --- | --- | --- | --- | --- | --- | --- |
| *Predictors* | *Estimates* | *CI* | *p* | *Estimates* | | *CI* | *p* | *Estimates* | | *CI* | *p* | *Estimates* | | *CI* | *p* | *Estimates* | | *CI* | *p* |
| (Intercept) | 25.48 | -11.01 – 61.96 | 0.168 | 23.51 | | -13.18 – 60.19 | 0.206 | 32.13 | | -3.29 – 67.54 | 0.075 | 25.69 | | -10.03 – 61.41 | 0.156 | 24.26 | | -11.00 – 59.52 | 0.175 |
| Group [A] | -1.92 | -24.90 – 21.07 | 0.868 | 1.96 | | -20.49 – 24.41 | 0.862 | -0.60 | | -22.60 – 21.40 | 0.957 | -2.22 | | -24.14 – 19.69 | 0.840 | 0.27 | | -21.85 – 22.38 | 0.981 |
| Group [B] | 16.87 | -5.99 – 39.74 | 0.146 | 20.87 | | -1.41 – 43.16 | 0.066 | 18.70 | | -3.16 – 40.55 | 0.092 | 16.57 | | -5.20 – 38.34 | 0.134 | 19.12 | | -2.88 – 41.12 | 0.087 |
| Group [C] | -6.21 | -29.15 – 16.73 | 0.591 | -2.36 | | -24.77 – 20.06 | 0.835 | -5.00 | | -26.96 – 16.96 | 0.651 | -6.51 | | -28.38 – 15.36 | 0.555 | -4.04 | | -26.11 – 18.03 | 0.716 |
| lFT pre | 0.84 | 0.69 – 0.99 | **<0.001** | 0.83 | | 0.68 – 0.98 | **<0.001** | 0.80 | | 0.66 – 0.95 | **<0.001** | 0.84 | | 0.69 – 0.99 | **<0.001** | 0.84 | | 0.69 – 0.98 | **<0.001** |
| **Random Effects** | | | | | | | | | | | | | | | | | | |  |
| σ^2^ | 355.13 | | | | 347.54 | | | | 429.87 | | | | 346.25 | | | | 347.92 | |  |
| τ_00_ | 406.00 _Subject_ | | | | 422.10 _Subject_ | | | | 356.16 _Subject_ | | | | 393.94 _Subject_ | | | | 401.70 _Subject_ | |  |
| ICC | 0.53 | | | | 0.55 | | | | 0.45 | | | | 0.53 | | | | 0.54 | |  |
| N | 39 _Subject_ | | | | 40 _Subject_ | | | | 40 _Subject_ | | | | 40 _Subject_ | | | | 40 _Subject_ | |  |
| Observations | 78 | | | | 80 | | | | 80 | | | | 80 | | | | 80 | |  |
| Marginal R^2^ / Conditional R^2^ | 0.736 / 0.877 | | | | 0.728 / 0.877 | | | | 0.712 / 0.842 | | | | 0.736 / 0.877 | | | | 0.736 / 0.878 | |  |

**Supplementary Table 7.** Sensitivity analysis for right Finger Tapping (FT) Speed.

|  | **No imputation** | | | | **PMM** | | | | **Random sample** | | | | **Unconditional mean imputation** | | | | **Bayesian Linear Regression** | | | |
| --- | --- | --- | --- | --- | --- | --- | --- | --- | --- | --- | --- | --- | --- | --- | --- | --- | --- | --- | --- | --- |
| *Predictors* | *Estimates* | *CI* | *p* | *Estimates* | | *CI* | *p* | *Estimates* | | *CI* | *p* | *Estimates* | | *CI* | *p* | *Estimates* | | *CI* | *p* |  |
| (Intercept) | 56.67 | 30.75 – 82.58 | **<0.001** | 56.79 | | 31.47 – 82.11 | **<0.001** | 57.69 | | 32.56 – 82.81 | **<0.001** | 56.81 | | 31.53 – 82.10 | **<0.001** | 55.14 | | 31.68 – 78.60 | **<0.001** |  |
| Group [A] | 6.84 | -14.68 – 28.35 | 0.528 | 8.14 | | -12.37 – 28.64 | 0.432 | 6.82 | | -13.71 – 27.34 | 0.510 | 6.61 | | -13.87 – 27.10 | 0.522 | 8.35 | | -12.53 – 29.24 | 0.428 |  |
| Group [B] | 19.55 | -1.85 – 40.95 | 0.073 | 20.94 | | 0.60 – 41.28 | **0.044** | 19.59 | | -0.80 – 39.99 | 0.059 | 19.32 | | -1.01 – 39.66 | 0.062 | 21.06 | | 0.23 – 41.90 | **0.048** |  |
| Group [C] | -20.84 | -42.75 – 1.07 | 0.062 | -19.43 | | -40.28 – 1.42 | 0.067 | -20.78 | | -41.69 – 0.13 | 0.051 | -21.07 | | -41.92 – -0.22 | **0.048** | -19.33 | | -40.69 – 2.04 | 0.076 |  |
| rFT pre | 0.67 | 0.56 – 0.77 | **<0.001** | 0.66 | | 0.55 – 0.76 | **<0.001** | 0.66 | | 0.55 – 0.77 | **<0.001** | 0.67 | | 0.56 – 0.77 | **<0.001** | 0.67 | | 0.56 – 0.77 | **<0.001** |  |
| **Random Effects** | | | | | | | | | | | | | | | | | | | | |
| σ^2^ | 322.49 | | | | 317.75 | | | | 359.46 | | | | 314.42 | | | | 331.74 | | | |
| τ_00_ | 341.72 _Subject_ | | | | 332.16 _Subject_ | | | | 309.00 _Subject_ | | | | 331.48 _Subject_ | | | | 327.32 _Subject_ | | | |
| ICC | 0.51 | | | | 0.51 | | | | 0.46 | | | | 0.51 | | | | 0.50 | | | |
| N | 39 _Subject_ | | | | 40 _Subject_ | | | | 40 _Subject_ | | | | 40 _Subject_ | | | | 40 _Subject_ | | | |
| Observations | 78 | | | | 80 | | | | 80 | | | | 80 | | | | 80 | | | |
| Marginal R^2^ / Conditional R^2^ | 0.778 / 0.892 | | | | 0.776 / 0.890 | | | | 0.771 / 0.877 | | | | 0.778 / 0.892 | | | | 0.795 / 0.897 | | | |

**Supplementary Table 8.** Sensitivity analysis for left Cortical Silent Period (lCSP).

|  | **No imputation** | | | | **PMM** | | | | **Random sample** | | | | **Unconditional mean imputation** | | | | **Bayesian Linear Regression** | | | |
| --- | --- | --- | --- | --- | --- | --- | --- | --- | --- | --- | --- | --- | --- | --- | --- | --- | --- | --- | --- | --- |
| *Predictors* | *Estimates* | *CI* | *p* | *Estimates* | | *CI* | *p* | *Estimates* | | *CI* | *p* | *Estimates* | | *CI* | *p* | *Estimates* | | *CI* | *p* |  |
| (Intercept) | 37.72 | -0.70 – 76.14 | 0.054 | 47.32 | | 11.70 – 82.95 | **0.010** | 48.79 | | 11.45 – 86.14 | **0.011** | 39.16 | | 1.85 – 76.47 | **0.040** | 50.12 | | 13.05 – 87.19 | **0.009** |  |
| Group [A] | 20.18 | -4.81 – 45.17 | 0.112 | 16.08 | | -8.27 – 40.42 | 0.192 | 14.35 | | -10.55 – 39.26 | 0.255 | 18.89 | | -5.01 – 42.79 | 0.119 | 14.24 | | -10.69 – 39.18 | 0.259 |  |
| Group [B] | -3.12 | -27.60 – 21.35 | 0.800 | -7.21 | | -31.02 – 16.61 | 0.548 | -8.93 | | -33.29 – 15.43 | 0.467 | -4.41 | | -27.78 – 18.97 | 0.708 | -9.03 | | -33.43 – 15.36 | 0.463 |  |
| Group [C] | 28.05 | 3.08 – 53.02 | **0.028** | 24.01 | | -0.33 – 48.36 | 0.053 | 22.29 | | -2.61 – 47.19 | 0.079 | 26.77 | | 2.89 – 50.65 | **0.029** | 22.19 | | -2.74 – 47.13 | 0.080 |  |
| lCSP pre | 0.72 | 0.47 – 0.96 | **<0.001** | 0.67 | | 0.43 – 0.91 | **<0.001** | 0.68 | | 0.43 – 0.92 | **<0.001** | 0.72 | | 0.47 – 0.96 | **<0.001** | 0.67 | | 0.42 – 0.91 | **<0.001** |  |
| **Random Effects** | | | | | | | | | | | | | | | | | | | | |
| σ^2^ | 576.24 | | | | 562.17 | | | | 562.40 | | | | 561.83 | | | | 562.14 | | | |
| τ_00_ | 406.42 _Subject_ | | | | 425.51 _Subject_ | | | | 457.96 _Subject_ | | | | 397.13 _Subject_ | | | | 460.34 _Subject_ | | | |
| ICC | 0.41 | | | | 0.43 | | | | 0.45 | | | | 0.41 | | | | 0.45 | | | |
| N | 39 _Subject_ | | | | 40 _Subject_ | | | | 40 _Subject_ | | | | 40 _Subject_ | | | | 40 _Subject_ | | | |
| Observations | 78 | | | | 80 | | | | 80 | | | | 80 | | | | 80 | | | |
| Marginal R^2^ / Conditional R^2^ | 0.438 / 0.670 | | | | 0.423 / 0.671 | | | | 0.405 / 0.672 | | | | 0.436 / 0.670 | | | | 0.402 / 0.671 | | | |

**Supplementary Table 9.** Sensitivity analysis for right Cortical Silent Period (lCSP).

|  | **No imputation** | | | | **PMM** | | | | **Random sample** | | | | **Unconditional mean imputation** | | | | **Bayesian Linear Regression** | | | |
| --- | --- | --- | --- | --- | --- | --- | --- | --- | --- | --- | --- | --- | --- | --- | --- | --- | --- | --- | --- | --- |
| *Predictors* | *Estimates* | *CI* | *p* | *Estimates* | | *CI* | *p* | *Estimates* | | *CI* | *p* | *Estimates* | | *CI* | *p* | *Estimates* | | *CI* | *p* |  |
| (Intercept) | 45.23 | 3.08 – 87.38 | **0.036** | 67.35 | | 25.92 – 108.78 | **0.002** | 48.28 | | 7.60 – 88.96 | **0.021** | 46.55 | | 5.79 – 87.30 | **0.026** | 60.19 | | 18.23 – 102.14 | **0.006** |  |
| Group [A] | 16.77 | -12.80 – 46.33 | 0.262 | 8.29 | | -22.75 – 39.34 | 0.596 | 19.92 | | -8.60 – 48.44 | 0.168 | 15.62 | | -12.65 – 43.89 | 0.274 | 9.27 | | -20.93 – 39.47 | 0.543 |  |
| Group [B] | 2.11 | -27.92 – 32.15 | 0.889 | -9.92 | | -40.66 – 20.82 | 0.522 | 3.65 | | -25.23 – 32.53 | 0.802 | 0.92 | | -27.75 – 29.60 | 0.949 | -7.33 | | -37.56 – 22.90 | 0.630 |  |
| Group [C] | 21.44 | -8.08 – 50.95 | 0.152 | 12.32 | | -18.54 – 43.18 | 0.429 | 24.30 | | -4.15 – 52.75 | 0.093 | 20.28 | | -7.92 – 48.49 | 0.156 | 13.59 | | -16.48 – 43.66 | 0.371 |  |
| rCSP pre | 0.70 | 0.44 – 0.96 | **<0.001** | 0.61 | | 0.34 – 0.87 | **<0.001** | 0.66 | | 0.40 – 0.91 | **<0.001** | 0.70 | | 0.44 – 0.96 | **<0.001** | 0.65 | | 0.38 – 0.92 | **<0.001** |  |
| **Random Effects** | | | | | | | | | | | | | | | | | | | | |
| σ^2^ | 634.04 | | | | 638.18 | | | | 713.36 | | | | 618.19 | | | | 632.18 | | | |
| τ_00_ | 656.80 _Subject_ | | | | 811.26 _Subject_ | | | | 608.66 _Subject_ | | | | 639.64 _Subject_ | | | | 760.45 _Subject_ | | | |
| ICC | 0.51 | | | | 0.56 | | | | 0.46 | | | | 0.51 | | | | 0.55 | | | |
| N | 39 _Subject_ | | | | 40 _Subject_ | | | | 40 _Subject_ | | | | 40 _Subject_ | | | | 40 _Subject_ | | | |
| Observations | 78 | | | | 80 | | | | 80 | | | | 80 | | | | 80 | | | |
| Marginal R^2^ / Conditional R^2^ | 0.448 / 0.729 | | | | 0.379 / 0.727 | | | | 0.420 / 0.687 | | | | 0.448 / 0.729 | | | | 0.402 / 0.728 | | | |
